# Unique or not unique? Comparative genetic analysis of bacterial O-antigens from the Oxalobacteraceae family

**DOI:** 10.18699/VJGB-22-98

**Published:** 2022-12

**Authors:** S.D. Afonnikova, A.S. Komissarov, P.D. Kuchur

**Affiliations:** Institute of Cytology and Genetics of the Siberian Branch of the Russian Academy of Sciences, Novosibirsk, Russia Novosibirsk State University, Novosibirsk, Russia; ITMO University, SCAMT Institute, St. Petersburg, Russia; ITMO University, SCAMT Institute, St. Petersburg, Russia

**Keywords:** O-antigen gene clusters, lipopolysaccharide genes, comparative analysis, O-antigen, Oxalobacteraceae, Massilia, Collimonas, Janthinobacterium, saccharide gene cluster, кластеры генов О-антигена, гены липополисахарида, сравнительный анализ, О-антиген, Oxalobacteraceae, Massilia, Collimonas, Janthinobacterium, кластеры генов сахаридов

## Abstract

Many plants and animals have symbiotic relationships with microorganisms, including bacteria. The interactions between bacteria and their hosts result in different outcomes for the host organism. The outcome can be neutral, harmful or have beneficial effects for participants. Remarkably, these relationships are not static, as they change throughout an organism’s lifetime and on an evolutionary scale. One of the structures responsible for relationships in bacteria is O-antigen. Depending on the characteristics of its components, the bacteria can avoid the host’s immune response or establish a mutualistic relationship with it. O-antigen is a key component in Gram-negative bacteria’s outer membrane. This component facilitates interaction between the bacteria and host immune system or phages. The variability of the physical structure is caused by the genomic variability of genes encoding O-antigen synthesis components. The genes and pathways of O-polysaccharide (OPS) synthesis were intensively investigated mostly for Enterobacteriaceae species. Considering high genetic and molecular diversity of this structure even between strains, these findings may not have caught the entire variety possibly presented in non-model species. The current study presents a comparative analysis of genes associated with O-antigen synthesis in bacteria of the Oxalobacteraceae family. In contrast to existing studies based on PCR methods, we use a bioinformatics approach and compare O- antigens at the level of clusters rather than individual genes. We found that the O-antigen genes of these bacteria are represented by several clusters located at a distance from each other. The greatest similarity of the clusters is observed within individual bacterial genera, which is explained by the high variability of O-antigens. The study describes similarities of OPS genes inherent to the family as a whole and also considers individual unique cases of O-antigen genetic variability inherent to individual bacteria

## Abstract (ru)

Многие растения и животные способны устанавливать симбиотические взаимоотношения с микроорганизмами, в том числе с бактериями. Специфика этих взаимодействий может приводить к разным последствиям для организма-хозяина. Взаимоотношения могут быть нейтральными, негативными либо выгодными для одной или обеих сторон. Примечательно, что взаимоотношения бактерия-хозяин не являются статичными: они могут изменяться в течение жизни организмов и в ходе их эволюции. Одной из структур, определяющих направление изменчивости, является О-антиген. В зависимости от особенностей его компонентов бактерия может избегать иммунного ответа со стороны организма-хозяина, становясь патогеном, либо устанавливать с хозяином мутуалистические отношения. О-антиген – это ключевой компонент наружной мембраны грамотрицательных бактерий. Этот компонент обеспечивает взаимодействие между бактериями и иммунной системой хозяина или фагами. Вариабельность структуры О-антигенов тесно связана с изменчивостью генов, кодирующих компоненты его синтеза. Гены и пути синтеза О-антигенов наиболее детально изучены у бактерий из семейства Enterobacteriaceae. С учетом высокого генетического и молекулярного разнообразия этой структуры даже между штаммами эти результаты могут не отражать все разнообразие О-антигенов, представленное у немодельных видов. В настоящей работе проведен сравнительный анализ генов, участвующих в синтезе О-антигена, для бактерий из семейства Oxalobacteraceae. В отличиe от существующих исследований, преимущественно основанных на методе ПЦР, в нашей работе использован биоинформатический подход, а сравнение проведено не на уровне одиночных генов, а на уровне кластеров. Мы обнаружили, что в случае Oxalobacteraceae генетическая организация О-антигена представлена несколькими кластерами, находящими- ся на значительном удалении друг от друга в геноме бактерий. Наибольшее сходство кластеров наблюдалось внутри отдельных родов бактерий, что объясняется высокой изменчивостью О-антигенов. В работе описано сходство генов О-антигенов, присущее семейству в целом, а также рассмотрены отдельные уникальные слу- чаи изменчивости их генетической структуры у отдельных бактерий.

## Introduction

The Oxalobacteraceae family belongs to the Burkholderiales order of Proteobacteria. According to the Integrated Taxonomic Information System (www.itis.gov) this family includes 55 verified species of 12 genera. Members of the Oxalobacteraceae family are stained negatively by Gram and presented in a wide range of habitats (outlined in Supplementary Materials, Table S1)1. Species were found in soils, including grassland, volcanic and heavy metal polluted soils, in water and glaciers (Baldani et al., 2014). Some of them are free-living, others may form various relationships with plants. Symbiotic species (Massilia, Herbaspirillum) are known to exhibit plant growth-promoting features, and can be beneficial in agriculture (Ofek et al., 2012; Peta et al., 2019; Grillo-Puertas et al., 2021). Occasionally, these relationships lead to plant diseases, for example, red stripe and mottle stripe diseases (Tuleski et al., 2020). The negative effect depends on the environment conditions. Examples of opportunistic features are described for Janthinobacterium and Herbaspirillum genera. Some species can be found in clinical samples and act as opportunistic pathogens for humans (Dhital et al., 2020).

Supplementary Materials are available in the online version of the paper: https://doi.org/10.5281/zenodo.7410337.


Beneficial effects from Oxalobacteraceae bacteria are related to agriculture and medicine. Farming industry utilizes these bacteria to improve plant growth. Mutualistic bacteria facilitate nitrogen assimilation to increase crops productivity. In medicine, bacterial lipopolysaccharides (LPS) can be used for vaccine development. This modern medicine development is called glycoconjugate vaccines. The methodology is already verified on the members of Enterobacteraceae family (Bazhenova et al., 2021) and can be scaled to other bacteria. Beyond vaccines, information related to LPS lies in biosensor systems. Systems are able to identify bacteria in samples based on their LPS composition, in particular O-antigens (Sannigrahi et al., 2020).

O-antigen became a convenient feature for serotyping due to its variability. Diversity of the oligopolysaccharide units and the selection of the host immune system directed at them highly contribute to the variability of O-antigens. In addition to this selection, there is the bacteriophage effect on the bacterial cell (Xi et al., 2019). All these factors explain the emergence of different serotypes within the same bacterial species

O-antigen is one part of bacterial LPS. Lipopolysaccharides are a specific structures (plural form) binding to the outer membrane of Gram-negative bacteria. It consists of three parts that are linked to each other in a particular order: phospholipid anchored to the membrane (lipid A or endotoxin), core region and O-antigen repeats. Lipid A is the hydrophobic domain anchoring LPS in the membrane. In chemical structure, lipid A is a phospholipid based on glucosamine. It forms the monolayer of the outer membrane. Lipid A is responsible for the toxicity of Gram-negative bacteria. The second component of LPS is the core part. The first and the second LPS components are synthesized on the cytoplasmic side of the inner membrane of the bacterial cell, after which they are transported by ABC transporters into the periplasmic space (Valvano, 2015). The third component of LPS is O-antigen, which is synthesized separately from the previous parts. In a periplasmic space, all parts of LPS are combined together, then the fully synthesized LPS is transported to the outer leaflet of the cell membrane (Doerrler, 2006).

The composition of LPS and its parts varies between different species and between strains (Caroff, Karibian, 2003). In some strains O-antigen can be absent, thus referred to as “rough” LPS, others containing it are “smooth” (Erridge et al., 2002). The O-antigen consists of a series of repeating oligosaccharide units. The length and composition of the monomers vary quite widely among strains (Perepelov et al., 2009). Repeats can be homodimers or heterodimers. In addition, units can be linked linearly or can create a branched structure (Liu et al., 2020).

Sugar nucleotides are basic molecules that form an O-antigen backbone. The most common can be divided into several groups: • dTDP-sugars (rfb/rml genes); • CDP-sugars (ddh genes); • GDP-sugars (man genes, gmd, col ); • UDP-glucoses (ugd, gla, galE ); • UDP-N-acetylglucosamines (gne, gna, fnl and mna genes).

Other nucleotide sugar genes include nna genes (N-acetylneuraminic acid synthesis), hdd genes and gmh (LD-mannoheptose and DD-manno-heptose) and dmh genes of 6-deoxy- D-manno-heptose synthesis pathway (Samuel, Reeves, 2003). The O-antigen chain is assembled via glycosyltransferases, which are responsible for combinations of sugar nucleotides.

The mechanisms of generating O-antigen and flipping are described in two variants: Wzy-dependent pathway and ABC-transporter pathway. The former is predominant among better-characterized O-antigens. A third variant is the synthasedependent pathway. Unfortunately, it is poorly described and has been observed rarely, for instance, in Salmonella species (Kalynych et al., 2014).

The initiation of all O-antigen synthesis pathways is a transfer of a sugar monophosphate to the undecaprenyl phosphate (Und-P) molecule, resulting in sugar-pyrophosphate-undecaprenyl (sugar-Und-PP). Sugar-Und-PP is able to accept further glycosylation reactions (Kalynych et al., 2014).

Uniquely to the Wzy-dependent pathway, Und-P-linked units are polymerized by Wzy (wzy gene) and subsequently flipped via Wzx (wzx gene). The chain length is controlled by Wzz protein (wzz). The completed structure is ligated to the outer core region via WaaL O-antigen ligase encoded by waaL (rfaL) gene (Han et al., 2012).

On the contrary, the ABC-transporter pathway needs only a single initiation reaction per O-antigen chain. Moreover, the entire polymerization process via glycosyltransferases is carried out in the cytoplasm. Then the completely generated O-antigen-Und-PP molecule is flipped to the periplasmic space by an ABC transporter, which is encoded by wzt and wzm genes. Similarly to the previously characterized pathway, the O-antigen ligase protein WaaL connects it to the core-lipid A (Samuel, Reeves, 2003).

In view of the above described, O-antigen becomes a highly variable structure. This feature makes the O-antigen attractive to a wide range of researchers. Nevertheless, there are rather few studies on comparative analysis of O-antigens and their genetic structure between bacteria at the family level. Most publications are devoted to single pathogenic or potentially pathogenic bacteria and avoid features of free-living or mutualistic species.

Detection and study of O-antigens have been made possible by the emergence of several methods involving both experimental and bioinformatics analysis of bacterial data. One of the traditional methods belonging to the first group is the bacterial glycotyping method based on the somatic antigen. In 2020, E.T. Sumrall et al. (2020) proposed a new method for quantitative separation of O-antigens. It is based on the use of a set of recombinant proteins that can interact with bacterial envelope receptors and domains. Bacterial O- antigens can also be detected by serological and agglutination test methods using sera specific to somatic antigens (Thakur et al., 2018). Another way to study O-antigen composition is the polymerase chain reaction method, which is widely used to compare O-antigens in several bacteria.

The emergence and subsequent decrease in the cost of sequencing opened new ways of O-antigen studying. In silico analysis methods have significantly reduced the time required for data processing, and many routine processes have been automated. Extensive databases have appeared that lead to the O- antigens analysis of several bacteria at once. In comparison to traditional methods of O-antigen detection, in silico methods are able to revise taxonomy misunderstandings, identify more genes related to O-antigen biosynthesis and evaluate their environment in a short time. Predicted features can be then verified by traditional laboratory methods. On the example of an Oxalobacteraceae member called Janthinobacterium sp. SLB01 (Belikov et al., 2021), the taxonomy was revised by this combined approach.

Here we present comparative analysis of O-antigens for 20 genomes from the Oxalobacteraceae family. According to the query in UniprotKB “(protein_name: O-antigen) AND (taxonomy_ id:75682)” there are only 456 genes whose proteins are annotated as O-antigen biosynthesis genes for this family. Our bioinformatics approach based on homologues search eliminates difficulties in gene annotation. We also shift from describing single genes to comparing O- antigens at the level of their candidate gene clusters to broad information about the gene content of Oxalobacteraceae O-antigens.

## Materials and methods

Data. Initial data was derived from NCBI databases and included 20 genomes. The main criterion of assembly selection was a rather high quality, that is, no more than ten contigs. The reason for such a criterion was to decrease the possibility of gene clusters being disrupted by unresolved sequences. Overall, we selected two Collimonas species (C. arenae and C. fungivorans), one species of genera Herminiimonas (H. arsenitoxidans), Oxalobacter (O. formigenes), and Undibacterium (U. parvum), four Janthinobacterium (J. agaricidamnosum, J. lividum, J. svalbardensis, J. tructae), two Oxalicibacterium (O. faegigallinarum and O. flavum) species and nine Massilia (M. albidiflava, M. armeniaca, M. flava, M. oculi, M. plicata, M. putida, M. timonae, M. umbonata, M. violaceinigra). Their RefSeq assembly accessions are presented in Supplementary Materials, Table S2.

Quality control and annotation. All 20 assemblies were additionally analyzed using QUAST tool, version 5.0.2 (Gurevich et al., 2013). The acceptable threshold number of contigs and scaffolds was eleven, only genomes with a lower number were selected. To obtain the most precise annotation, we used two annotation tools, Prokka version 1.14.6 (Seemann, 2014) and eggNOG version 2.1.6 (Huerta-Cepas et al., 2019).

Putative O-antigen genes search. Searching for genes coding components for O-antigen synthesis and processing based on their names was unproductive because of the abundance of various synonymous tags. Therefore, we used an approach based on orthology. All O-antigen related genes for Escherichia coli strains described in the paper (Iguchi et al., 2015) were obtained with their amino acid sequences and used as reference. We also added genes from the KEGG database, a pathway of O-antigen synthesis for E. coli https://www. genome.jp/pathway/ecoi00541. We additionally analyzed the O-antigen ligase gene rfaL (waaL), because it was shown that O-antigen may be absent in some bacteria (Kime et al., 2016). As waaL is essential for final stages of O-antigen processing for the majority of bacteria, its absence may be associated with a lack of OPS on the cell wall (Wang et al., 2010). This data consisted of gene sets for each serogroup and approximately 420 unique genes in total (Supplementary Materials, Table S3).

In order to find unique genes among this data, sequences were clustered using UCLUST (Edgar, 2010) algorithm with the usearch32 tool, with threshold identity > 0.4. The reason for the rather low threshold was the excessive amount of clusters at higher numbers, mainly because of high gene variation. For the next step, we chose centroids of each cluster as representative sequences of 27,000 bp in length (Cimermancic et al., 2014). However, another important parameter for our definition is genes on borders. Thus, for an array of three genes, if genes on the borders of the set coincide, we also define this set as a cluster. A more detailed investigation of the obtained gene clusters with respect to their structure, function and sequence similarity was conducted using eggNOG and BLAST (v.2.5.0+) tools

Verification of the identified candidate genes was performed via functional Pfam domains search (Supplementary Materials, Table S4). Lists of domains were obtained manually, from (Iguchi et al., 2015; Pereira et al., 2015). The HMMER software hmmer.org version 3.3.2 allowed the detection of those domains in FASTA amino acid sequences of all genomes. Some genes were checked manually using online Pfam sequence search https://pfam.xfam.org (Mistry et al., 2021). Characterization of genes shown to be uninvolved in O-antigen biosynthesis processes was performed using KEGG databases (Kanehisa, 2000).

Phylogenetic tree reconstruction. Phylogenetic tree was constructed to explore evolutionary relationships between the chosen Oxalobacteraceae taxa. Several species of the Burkholderaceae family were selected to create an outgroup (Burkholderia sordidicola, B. unamae, B. symbiotica, Ralstonia pickettii, Cupriavidus necator). 16S rRNA sequences for 13 Oxalobacteraceae species were derived from published papers (Lim et al., 2003; Caballero-Mellado et al., 2004; Zhang et al., 2006; Sheu et al., 2012; Baldani et al., 2014; Koh et al., 2017; Daniel et al., 2021; Jung et al., 2021). Barrnap version 0.9 (RRID:SCR_015995) was used for seven other genomes (C. arenae, C. fungivorans, J. agaricidamnosum, J. lividum, J. svalbardensis, M. timonae, O. flavum) to derive 16S rRNA sequences (Supplementary Materials, Table S5).

16S rRNA sequences were aligned using R-coffee, the T-coffee web-server RNA sequences alignment tool (Notredame et al., 2000). This tool takes into consideration the RNA secondary structure. Default multiple alignment options were chosen. The resulting alignment was used for constructing a phylogenetic tree using IQ-TREE web server (Nguyen et al., 2015). DNA was selected for sequence type, other options remained default. The best-fit model was TN+F+I+G4, the tree constructed with the Maximum likelihood method. Consensus tree was constructed from 1000 bootstrap trees and branch lengths were optimized by Maximum likelihood on original alignment. The results were visualized using Archaeopteryx 0.9928 (Han, Zmasek, 2009).

**Fig. 1.**
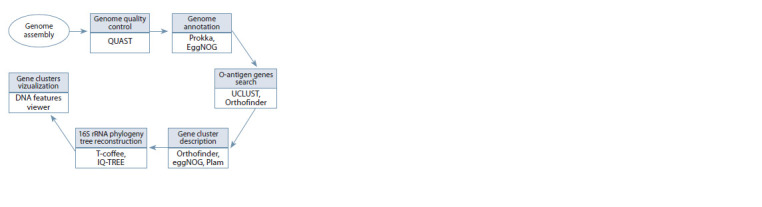
Schematic representation of the data analysis algorithm used in the current study.

Gene clusters visualization. To visualize the found clusters we developed a Python script based on the DnaFeaturesViewer library (https://edinburgh-genome-foundry.github.io/DnaFeaturesViewer/ index.html#more-biology-software). The code is available on this page https://github.com/svetaafonnikova/ O-antigen-project/blob/main/draw_cluster.py. All steps of the data analysis algorithm are schematically depicted in Fig. 1.

## Results

Assembly quality characterization

Out of all 20 assemblies, 15 were at the level of complete genomes. M. timonae assembly consisted of a single contig with N50 equal to the length of this contig. Two assemblies contained plasmid sequences (M. putida and M. violaceinigra). Another pair, O. faecigallinarum and O. flavum, contained ten and nine contigs, respectively.

Using IGV (v. 2.11.1) (Robinson et al., 2011) we confirmed that the identified O-antigen gene clusters were not located on plasmid fragments in case of plasmid containing genome assemblies. Secondly, O-antigen gene clusters were not situated on the borders of contigs, thus any breaks inside clusters were excluded.

Description of gene clusters

In general, almost all of the analyzed species contained more than two O-antigen gene clusters. These clusters are scattered around the genome and include not only O-polysaccharide genes, but genes of other functions. The visualization for all 20 species can be found in Supplementary Materials, Fig. S1. In the text below, we will describe these clusters for each genus used in the study.

Collimonas. In both C. arenae and C. fungivorans we detected O-antigen ligase gene rfaL (or waaL) immediately adjacent to galE gene involved in nucleotide sugar synthesis. In addition, both genomes contain wzm and wzt genes. Furthermore, they share the same cluster with manB and wfaK on borders. All genes and their order coincide except one glycosyltransferase gene wbaS, absent in C. fungivorans.

Regarding other differences, the former species consists of three clusters, the latter consists of four. One of C. fungivorans clusters contains O-antigen unit synthesis (rmd, gmd, manC), processing genes (wzm, wzt) and a triplet of glycosyltransferase gene wbaX. Remarkably, in C. arenae these processing and unit synthesis genes are included in a single cluster with rfbABCD and manB genes on the borders.

Herminiimonas. According to our analysis, H. arsenitoxidans genome possesses three O-antigen gene clusters, with rfaL gene located outside all of them without any OPS genes beside. Regarding genes involved in processing, only wzx was observed. There are duplication instances for L-Rhamnose biosynthesis gene rfbD, sugar transferase genes wbaT and wbaS. One cluster contains a rather small number of genes we are interested in compared to not O-antigen ones. These unnecessary for OPS production genes partake in phosphate metabolism.

Janthinobacterium. J. lividum carries two clusters and J. agaricidamnosum comprises three gene clusters involved in O-antigen synthesis, whereas J. svalbardensis and J. tructae include four. The latter two share identical clusters with UDP-N-acetylglucosamine pathway, wbqA and wbqB on the one end and glycosyltransferase gene wbdH on the other. All but J. agaricidamnosum have duplications of rfbABCD genes. All four genes are duplicated in J. lividum and J. svalbardensis, J. tructae possesses three copies of rfbA and rfbB. Furthermore, the J. tructae cluster with rfbBA and fnlA borders is almost similar to a part of another larger O-antigen gene cluster. In J. lividum and J. svalbardensis we found a common OPS related gene cluster flanked by wbqB and wbhQ. This gene set includes dTDP-glucose pathway genes rfbABCD and vioA. Still, the latter species has glycosyltransferase wbaS next to wbqB, which J. lividum lacks in this position. To add, wzx gene was located after vioA in J. lividum, however, we didn’t observe any significant domains for J. svalbardensis in that position. The O-antigen ligase was observed in all genus members. It lies far from any depicted cluster.

Considering genes not included in our initial gene list, there are genes involved in LPS core synthesis (waaD), polysaccharide transport gene (wza), genes characteristic to O-antigen production in other bacteria species (rfbG, rfbF).

**Fig. 2.**
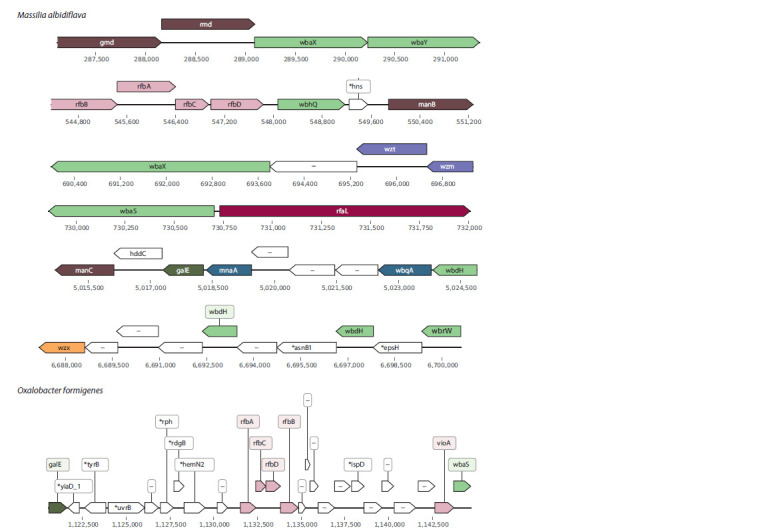
The O-antigen gene clusters from M. albidiflava and O. formigenes. Unannotated genes are designated as “–”. Сolors code for biosynthetic pathways. Orange genes are involved in Wzx/Wzy pathway, violet are involved in Wzm/Wzt pathway, rose genes partake in dTDP-sugar pathway, dark green, in UDP-sugar pathway, brown, in GDP-sugar pathway, rfaL gene is coded in red. UDP-N-Acetylglucosamine genes are blue, and transferase genes are light green. Genes involved in other pathways are white. The complete graphical visualization of OPS gene clusters for other analyzed species can be found in Supplementary Materials, Fig. S1.

Massilia. According to our analysis, Massilia is the genus with the highest number of O-antigen gene clusters. M. oculi has six clusters, M. flava, M. umbonata and M. violaceinigra possess only four and others contain five clusters (Fig. 2).

We observed some patterns in gene clusters between species. All Massilia species carry the gmd_rmd_wbaX_wbaY (in exact order) cluster. Only in M. oculi and M. timonae rfbBDAC genes (order in cluster) are presented as an independent cluster. In other genomes, these genes are surrounded by various O-antigen related genes. The same cluster with rfbBACD (order in cluster) genes and manB occurs in M. violaceinigra, M. plicata, M. flava, M. armeniaca and M. albidiflava. A single gene unrelated to O-antigen production is DNA-binding protein gene hns.

To add more similarity between M. oculi and M. timonae, they possess identical clusters consisting of wbrW, wbdH, wbqB, ugd on the one end and wzx on the other end. Genes located among them partake in infection initiation (espH), amino acid biosynthesis (asnB1), acyl-CoA and fatty acids biosynthesis ( fadD).

In all assemblies, we observed wzm and wzt genes. Most of the species contain these genes in the order wzm, wzt, unannotated gene and wbaX. The group with such a set includes M. albidiflava, M. armeniaca, M. oculi, M. putida, M. plicata, and M. timonae. Another gene context is larger, the cluster is flanked by wzm/wzt and vioA. Between them are two glycosyltransferase genes wbaX with different lengths, unannotated genes and gtrB. The latter is a viral gene, and it can actually modify O-antigen structure. However, it was not described for the E. coli OPS gene cluster. Finally, in M. violaceinigra we found a unique set (not O-antigen biosynthesis gene cluster by our definition) of OPS processing genes and wbaX. There are three unannotated genes and two wzt. For the one beside wzm we didn’t verify a specific domain, it was indicated as a gene not involved in O-antigen synthesis. The domain structure for wzt laying further from wzt was proved.

One of M. armeniaca clusters contains a full cluster described for M. plicata. It starts with mnaA, proceeds with wbrW and three copies of wbdH. In the former species, wzx with unannotated genes is added after the third wbdH. M. umbonata shares the most part of this cluster with M. armeniaca, except it lacks mnaA at the beginning. All genomes except M. plicata include the galE, hddC and manC part in the exact order in one cluster per genome

Considering O-antigen ligase gene rfaL, in M. albidiflava, M. oculi, M. plicata, M. timonae and M. violaceinigra this gene is located next to wbaS. The rest of the species contain rfaL outside O-antigen clusters

It can be noticed that some genes, for instance, wbaS, rfbA and rfbB, mnaA, wbdH, are presented in two or more copies in genomes.

Oxalicibacterium. Three clusters were identified for each species of the Oxalicibacterium genus. They share a cluster flanked by wfaK and manC. Their content slightly diverges from each other. O. flavum has more genes, including an additional O-antigen related gene ugd. The OPS ligase gene rfaL was identified in both assemblies, however, they are located in different contexts.

Oppositely to O. flavum, O. faecigallinarum carries UDPN- Acetylglucosamine pathway genes ( fnlA, fnlB, mnaA, gne, wbqB). On top of it, in the O. faecigallinarum we could locate duplications of the rfbABCD part, lying in discrete clusters and ordered in a different manner. However, rfbD gene in the bigger cluster is rather dubious, the smaller length compared to other rfbD instances adds more uncertainty. We did not find this gene using Orthofinder analysis, although there is a Pfam domain corresponding to typical rfbD (RmlD_sub_bind) and it was annotated as rfbD by EggNOG.

We could detect wzt/wzm genes only in O. flavum assembly. The second species probably either does not carry these genes or they can be located outside clusters in unread spaces between contigs

Oxalobacter. For O. formigenes we identified a single OPS cluster carrying dTDP-sugar pathway genes rfbABCD and vioA and UDP-glucose synthesis gene galE. The rest of the genes in the cluster are involved in nucleotide metabolism and cofactor synthesis. Also, any O-antigen processing genes were undiscovered. We couldn’t detect rfaL gene in the given assembly. Moreover, even NCBI databases don’t have any information considering this gene or protein in the Oxalobacter genus

Undibacterium. For U. parvum two clusters were identified, wzt and wzm genes, were located outside them. Interestingly, wzt gene is smaller in comparison to this gene’s length in other Oxalobacteraceae species. Typical of them, wzt is longer than wzm by approximately 400 bp. In contrast, U. parvum’s wzt is almost the same size as wzm. Using Pfam service, the gene’s domain (ABC_tran) was verified.

Both clusters possess transferase and nucleotide sugar genes. Most spaces between OPS synthesis genes are unannotated genes, except dyp (peroxidase) and ansA (asparaginase) genes. The cluster carrying rfbABCD genes has a copy of manC gene and two wbaX genes, which have different sizes.

Phylogenetic tree

**Fig. 3.**
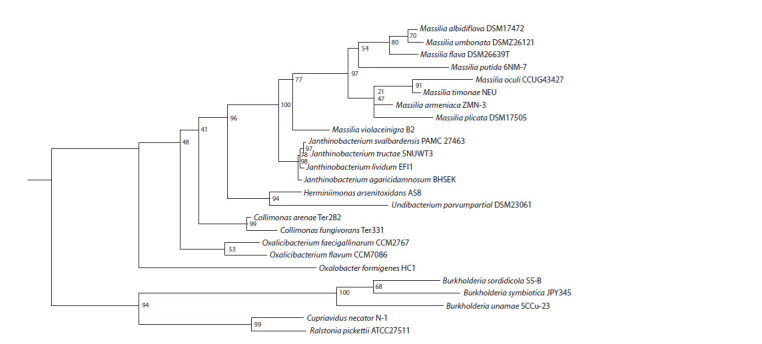
Phylogenetic reconstruction of Oxalobacterceae family members selected for the study based on 16S rRNA and created using Maximum likelihood method. The consensus tree was obtained from 1000 bootstrap trees. The sequence data is described in Supplementary Materials, Table S3.

The phylogenetic dendrogram based on 16S rRNA showed that the chosen species clustered together considering their genera (Fig. 3). There had been no study including all species and their exact strains used in the current work. Therefore, we could compare only some clades of the tree. Similar to other studies, the first species to branch off is Oxalobacter species. Contrary to literature reports, our tree has a distinct Oxalicibacterium group and Collimonas with the rest of the species of the Oxalobacteraceae family (Baldani et al., 2014). However, the bootstrap support is rather small at this node. The Janthinobacterium group formation coincided with other papers (Jung et al., 2021). Some Massilia species clustered according to literature (Feng et al., 2016; Ren et al., 2018). Also, we obtained an unresolved node between M. armeniaca and M. plicata. The gene lengths used in the analysis varied between 1400 and 1500 bp for most cases (see Supplementary Materials, Table S5).

## Discussion

In this work, we determined candidate genes involved in O- antigen biosynthesis in bacteria from the Oxalobacteraceae family. In comparison to well-studied E. coli O-antigen genes, they are presented in the form of several clusters. A similar situation has already been described for non-model bacteria (Hug et al., 2010). These clusters are dispersed across the genome. Clusters include O-antigen genes together with additional genes, which are necessary for LPS biosynthesis (for example, for core part synthesis and LPS parts binding) or partake in other processes. The E. coli O-antigen gene cluster was studied by traditional laboratory methods, in particular, by PCR (DebRoy et al., 2011; Iguchi et al., 2015). These methods aim to detect specific genes, whereas in silico methods take into account the gene environment. In other words, they allow structures to be studied at the cluster level. Thus, our approach helps to expand understanding of the O-antigen genetic composition in bacterial genomes

During OPS genetic structure comparison, we identified common features presented in all species inside the Oxalobacteraceae family. In particular, the group of rfbABCD genes was detected in each bacterium. The order of these genes varies, however, they are always placed together in one cluster. No one gene has deletions, nonsense mutations and other sequence abnormalities. According to the results, the studied bacteria should have a correct dTDP-rhamnose synthesis.

More similarities were found within each genus. These similarities relate mainly to individual genes or pairs of genes. A possible explanation lies in the high level of variability of O-antigens and the rate of bacterial mutations. O-antigens undergo changes so frequently that most of the similarities occur at the species or lower levels rather than at the genus or family level (Liu et al., 2008).

In 13 bacteria species, wzm and wzt genes were detected. We consider the Wzm-Wzt transporters pathway as the main path of O-antigen biosynthesis in this case (Wang et al., 2010). Wzx-Wzy pathway was not confirmed due to the absence of wzy genes.

Another interesting finding concerns gene duplication. The most repetitive genes were identified in Massilia species (see Fig. 2). Its O-antigens clusters may contain up to three copies of the same gene. We suggest two possible explanations. The first one is related to the biological features of LPS. The same gene can provide the synthesis of several parts of LPS. The appearance of additional gene copies can increase the amount of protein in the cell or maintain its level in case one of the gene copies is broken. The second explanation is linked with an algorithm of O-antigen genes search. In our approach, genes are detected according to the principle of homology, so similar genes can be assigned the same name.

Symbiotic bacteria Oxalobacter formigenes lacks O-antigen ligase gene (waaL) in O-antigen clusters, which may indicate the absence of O-antigen. The lack of the mentioned structure was discussed by J.K. Kim et al. (2016) for Burkholderia bacteria species. With our results, we confirm the possibility of loss of O-antigen genes in symbiotic bacterial species.

## Conclusion

Overall, the findings of this study indicate differences of nonmodel bacteria from the model one by the example of the Oxalobacteraceae family. We suggest that the characterized OPS gene cluster composition is atypical. So far, most papers, which explored these genes for other bacteria, described only a single gene cluster. The O-antigen genetics of non-model bacteria is highly diverse, which is proved by the bioinformatic approach. The search for homologous sequences allows us to expand and deepen our understanding of gene clusters involved in O-antigen biosynthesis. Further investigation of the Oxalobacteraceae O-antigen genetic composition can be confirmed by laboratory methods.

## Conflict of interest

The authors declare no conflict of interest.
